# Peripheral Neutritis in Tuberculosis

**Published:** 1887-03

**Authors:** 


### *PERIPHERAL NEURITIS IN TUBERCULOSIS.*

The following are the conclusions of a lengthy article by MM. Pitres and Vaillard in the *Rev. de Médecine*, March 10, 1886:

1. It is not infrequent to find, in the course of tuberculosis, as in that of other infectious diseases, the peripheral nerves becoming the seat of parenchymatous alterations, presenting the histological characters of the so-called degenerative neuritis.

2. These neurites develope locally, and are not at all dependent on pre-existing cerebral or spinal disease. They are met with in subjects whose nervous centres (brain, cord, meninges,) and the spinal root are in a perfectly healthy condition.

3. They may affect indifferently the sensory, motor, and the mixed nerves. They may also involve the cranial nerves (optic and aculomotor), or the pneumogastric and phrenic nerves, etc.

4. Their very complex and variable symptomatology is as ye imperfectly known. Nevertheless, by comparison of the facts published up to date, we may divide them into three groups.

The first of these groups comprises those cases in which the symptoms of neuritis revealed at the autopsy had passed unnoticed amongst the grave disorders dependant upon the evolutions of the tubercular disorder (latent neurites).

In the second group fall the cases in which localized or diffuse muscular atrophies have constituted the predominant symptoms (myotrophic neuritis).

In the third group, finally, those cases figure in which the neuritis has during life produced more or less serious sensory disorders, hyperæsthesias, anæsthesias, neuralgias, etc. (painful or anæsthetic neuritis).

5. The frequency of peripheral neuritis amongst tuberculous subjects, the variability of its distribution, and, in consequence, of its symptomatology, explains the clinical polymorphism of the majority of the nervous disorders which arise in the progress of tubercular disease.—*Neurological Review.*

